# The Old Preacher

**Published:** 1873-02

**Authors:** 


### THE OLD PREACHER.

A minister in good standing, who has been a worker in the Presbyterian church in Brooklyn, becoming no longer able to make a living by his profession, has been for the last five years the inmate of a poor house in Vermont. Doubtless the reader will feel indignant, that such an outrage should have been permitted in a denomination of christians which has done so much for the human race; for the destitute at home, and for the perishing and abroad; but very likely it will end in indignation or smoke, except in the hearts of a few who may consider the matter. The Trinity corporation gives a thousand dollars a year, or more, to all clergymen who become disabled while preaching for them. There are a number of insurance companies, especially at Hartford, Connecticut, who give their old and faithful servants and officers a retiring pension. But a clergyman ought to have it as a matter of right and not as a charity, when he has spent all his time in working for the church in any capacity. But this support in old age should not be left to any contingency; some plan should be devised by which such support could be relied upon as the result of a systematic arrangement. In the absence of anything better, it might answer a good purpose to adopt the following plan: when a community employs a clergyman let them have his life insured for five thousand dollars. in addition to his salary, with the provision, that on his arriving at a certain age, he should have a specified amount paid to him annually; or if he died, that amount should be paid to his family. Should he go to preach to another people, let them continue the arrangement; let this be considered as necessary a part to his "call," as a house to live in. Some plan of this kind would remove a burden from the minds of the clergy, which is of mountain weight, and they would be stimulated to work on with an alacrity which would otherwise be impossible.

